# A transcriptomic variation map provides insights into the genetic basis of *Pinus massoniana* Lamb. evolution and the association with oleoresin yield

**DOI:** 10.1186/s12870-020-02577-z

**Published:** 2020-08-13

**Authors:** Qinghua Liu, Yini Xie, Bin Liu, HuanhuanYin, Zhichun Zhou, Zhongping Feng, Yadong Chen

**Affiliations:** 1 Research Institute of Subtropical Forestry, Chinese Academy of Forestry, Hangzhou, 311400 Zhejiang People’s Republic of China; 2 Zhejiang Provincial Key Laboratory of Tree Breeding, Hangzhou, 311400 Zhejiang People’s Republic of China; 3 Sichuan Agricultural University, Chengdu, 611130 Sichuan People’s Republic of China; 4 Laoshan Forest Farm of Chunan County, Chunan, 311700 Zhejiang People’s Republic of China; 5 Biomarker Technologies Corporation, Beijing, 101300 People’s Republic of China

**Keywords:** SNP, Genetic diversity, Population structure, Geographic origin, Associative transcriptomics, *Pinus massoniana*

## Abstract

**Background:**

Masson pine *(Pinus massoniana* Lamb.), the dominant native coniferous species in southern China, is commercially important for supplying timber and oleoresin. However, knowledge of the genetic variability of masson pine germplasm is still limited. In this study, the genetic diversity and population structure of masson pine germplasm were assessed using 204 wild accessions from 10 main distribution regions using 94,194 core single-nucleotide polymorphisms (SNPs) obtained from transcriptome sequencing data.

**Results:**

The average expected heterozygosity was 0.2724, implying abundant genetic diversity within masson pine germplasm. Analysis of molecular variance (AMOVA) revealed that 3.29% of the variation was sourced from genetic differentiation. Structure analysis identified two geographically distinct groups. Discriminant analysis of principal components (DAPC) showed that one of those groups was further divided into two clusters. Sichuan and Chongqing provenance is the geographical origin, which diffused outward along two different lines. Oleoresin yield is reflected in the evolution of the two groups, and exhibits two different trends along the two lines of diffusion. The oleoresin yield may be associated with the genes of chitinase, CYP720B, cytochrome P450, ABC transporter, and AP2/ethylene-responsive transcription factor (ERF) based on SNPs and expression.

**Conclusions:**

SNP markers from transcriptome sequencing are highly capable of evaluating genetic diversity within different species, as well as the genetic control of objective traits. The functions of these genes will be verified in future studies, and those genes strongly associated with oleoresin yield will be used to improve yields by means of early genotype selection and genetic engineering.

## Background

As a dominant native tree species, masson pine (*Pinus massoniana* Lamb.) is a commercially important conifer for supplying timber and oleoresinin China. The natural distribution of this species extends from 21°41′N to 33°56′N and from 102°10′E to 123°14′E, with a planting area of 2 million hectares [1]. The provinces of Guangdong, Guangxi, Hu’nan, Sichuan, Chongqing, Guizhou, Zhejiang, Fujian and Jiangxi are the main natural distribution regions of masson pine in China [2]. Because the tree has the characteristics of rapid growth and tolerance to barren soil, it is often considered as a pioneer species for afforestation in areas of sparsely covered mountain. Genetic diversity is critical for the long-term survival of species, which drives species to adapt to various abiotic and biotic stresses in order to avoid extinction [3]. For most tree species, large genetic variations in growth, terpenoid yield, resistance to diseases, etc. can be observed between and within natural populations based on provenance or family analysis [4–6]. To discover the genetic variations among the main economic characteristics of masson pine, large-scale provenance experiments have been carried out in China since 1978. Two complete native-range provenance trails and many partial native-range provenance trails have been built in China [2], which have provided good materials to reveal the interplay and the significance of the various evolutionary forces giving rise to phenotype diversity and to formulate a gene conservation strategy that captures the natural genetic diversity within spieces. A classical pattern in the geographical variation with latitude has been found for diameter at breast height (DBH) in masson pine [7].

As a secondary substance obtained from masson pine, oleoresin is an important natural product that is used as the source for many different compounds in the chemical industry [8, 9], for protecting against insects and disease [10, 11], and for use as an advanced liquid biofuel [12]. Significant genetic variations related to the yield of oleoresin have also been observed among different families of masson pine, which ranged between 14.12 and 50.55 g per day [13, 14]. Zeng et al. and Westbrook et al. also reported that variation in oleoresin yield was heritable in loblolly pine (*P. taeda*) and could be increased 1.5- to 2.4-fold in one generation through selection.

Molecular markers are very useful in identifying germplasm, assessing biodiversity, and describing the geographic patterns of genetic variation. Single nucleotide polymorphisms (SNPs) are commonly used in genetic studies. Taking advantage of next generation sequencing (NGS) technologies, millions of SNPs for crops can be rapidly developed at low cost [9]. These high-throughput SNPs have been successfully used to evaluate genetic diversity and to deduce population structure [15, 16] and kinships [17]. As an important forest tree in southern China, a high-density SNP map is essential for genetic innovation and improving the traits of masson pine in future breeding programs. To date, however, there are no reports of the complete genome sequences for developing SNP markers to study the genetic diversity and structure of masson pine. Only partial masson pine germplasms have been analyzed using random amplification polymorphic DNA (RAPD) [18], inter-simple sequence repeat (ISSR) [19], simple sequence repeat (SSR) [20], and inter-retrotransposon amplified polymorphism (IRAP) [21].

Associative transcriptomics has contributed significantly to identifying sequence polymorphisms and transcript abundances linked to phenotypic variation, especially in non-model species [22, 23]. In addition, high-quality full-length transcripts are critical for functional assays and for understanding genetic diversity [24]. In this work, we first constructed high-quality transcript reference sequences through a combination of a full-length transcriptome and NGS-based unigenes. Then the RNA sequencing (RNA-Seq) of 204 representative accessions was adopted for de novo SNP discovery to generate a genome-wide variation map. The aims of our study were: (1) to assess the genetic diversity, population structure, and geographic origin of masson pine; and (2) to reveal the genes associated with oleoresin yield. The findings of our study will then be useful for managing this species and for expounding the mechanism of formation of high-yielding oleoresin.

## Results

### Sequencing and variation discovery

Research into and the breeding program of masson pine have been hampered by a lack of high-quality genome sequences because of its extremely large and complex genome, as presumed by that of the closely related species *P. taeda* [25]. To overcome this obstacle, we constructed a high-quality full-length transcript data set from the secondary xylem transcriptome of one mix sample using the PacBio Single Molecule, Real-Time (SMRT) Sequencing platform. A total of 81,837 high-quality and non-redundant full-length transcripts were obtained from 18 Gb of PacBio subreads. To explore the origins and patterns of genetic diversity, we also designed the population transcriptome experiments on the Illumina HiSeq™ 2000 sequencing platform for 204 geographically diverse masson pine genotypes, which had been collected from their main habitats in China; a total of 341,714 non-redundant unigenes were assembled. Following combination of the full-length transcripts and the unigenes, 423,288 non-redundant transcripts were considered as reference sequences for further analysis (Additional file 1: Table S1). On average, 85.02% of the reads for each sample were successfully mapped to the reference sequences, suggesting a high state of completeness of the reference transcripts (Additional file 2: Table S2).

A total of 1,326,230 SNPs and 153,459 insertions/deletions (InDels) were detected from the transcriptomes of the 204 genotypes using GATK packages [26], with an average SNP density of 3.13 per transcript (Additional file 3: Table S3). Among these SNPs, 94,194 core SNPs with a minor allele frequency (MAF) ≥ 0.05 and missing genotype calls < 5% were retained for further analysis, occupying 7.1% of the total set. These core SNPs included 23,864 (25.33%) non-synonymous (nsSNPs) (Additional file 4: Table S4). This transcriptome variation map will benefit core germplasm identification, genetic variation research, and artificial breeding.

### Genetic diversity of masson pine

The genetic diversity among *P. massoniana* germplasms from the main regions where the species is distributed was investigated based on 94,194 SNPs. The observed heterozygosity (*H*_*0*_) value was lower than the expected heterozygosity (*H*_*e*_) value for each population, ranging from 0.2211 (Guangxi) to 0.2358 (Sichuan and Chongqing). The *H*_*e*_ values were similar among the different populations, and ranged from 0.3011 (Jiangxi) to 0.3124 (Sichuan and Chongqing) (Table 1). The values of the inbreeding coefficient (*F* index) ranged from 0.2242 (Sichuan and Chongqing) to 0.2714 (Guangxi), with an average value for the overall population of 0.2731, indicating that the SNPs in the Sichuan and Chongqing population had the highest polymorphism. Putative differences among the nine populations were tested by AMOVA based on 94,194 SNPs (Table 2). The results showed that the differentiation among the populations was explained by 3.29% of the total variance. Only 0.01% of the variation was found among the different subpopulations, suggesting a closed kinship within them. In summary, our variant data set provides a comprehensive overview of the genomic diversity at various scales of population and represents a rich source of genetic information for exploitation by both the academic and the agricultural research communities.

**Table 1:** Summary of genetic variation statistics

Populations	H_0_	H_e_	MAF	F
Total	0.1849	0.2724	0.1870	0.2731
Guangdong	0.2285	0.3067	0.2166	0.2328
Guangxi	0.2211	0.3046	0.2147	0.2714
Guizhou	0.2351	0.3122	0.2209	0.2425
Sichuan and Chongqing	0.2358	0.3124	0.2248	0.2242
Anhui	0.2276	0.3064	0.2164	0.2320
Zhejiang	0.2239	0.3021	0.2123	0.2513
Jiangxi	0.2230	0.3011	0.2117	0.2521
Hunan	0.2291	0.3078	0.2168	0.2384
Fujian	0.2286	0.3068	0.2174	0.2290

H_0_, observed heterozygosity; H_e_, expected heterozygosity; MAF, minor allele frequency; F, inbreeding coefficient

**Table 2:** AMOVA of the variability of the clones from different populations of masson pine

Source of variation	Degree of freedom	Sum of square	Estimated variation	Percentage of variation	F-statistic	P-value
Among populations	8	55,488.43	93.20	3.29	*F*_*st*_	< 0.0001
Among clones within populations	195	533,808.76	0.41	0.01	*F*_*is*_	0.9218
Within clones	204	558,280.00	2736.67	96.69	*F*_*it*_	0.2395
Total	407	1,124,577.19				

### Construction of the *P. massoniana* core germplasm of high diversity of SNP

**Fig. 1:**
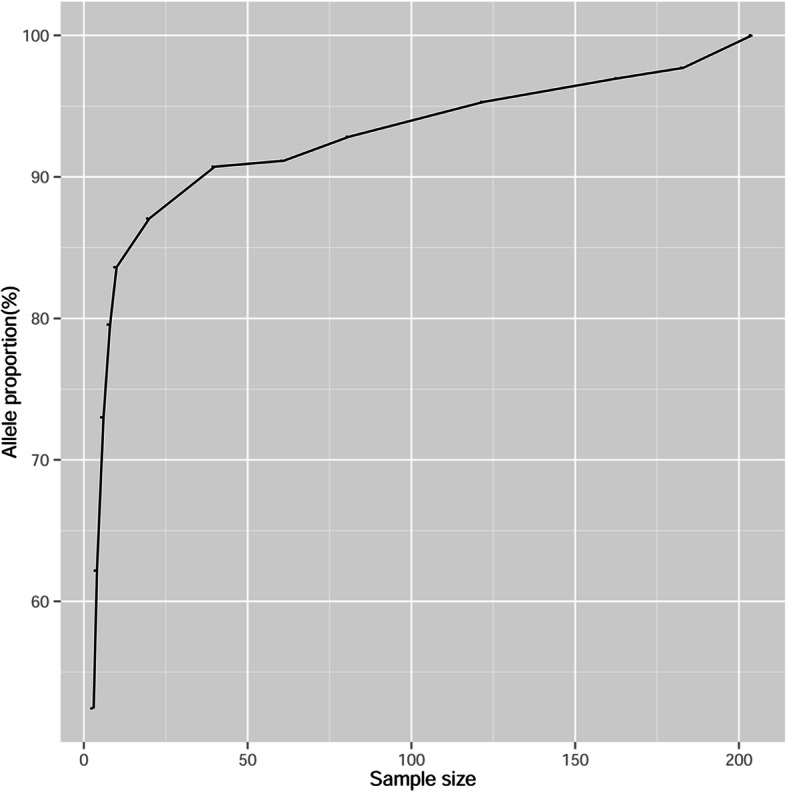
Core collection size with the proportion of alleles captured by SNP markers in masson pine

The allelic diversity among *P. massoniana* accessions can be maximized by SNP markers. The redundancy curve showed that masson pine allelic diversity can be represented by more than 40 core germplasms. These 40 representative genotypes, which accounted for only 20% of the collection, represented more than 90.7% of the allelic diversity (Fig. 1 and Table 3). Therefore, the minimum size of the core germplasm could constructed from these 40 representative accessions, including nine accessions from Zhejiang, nine accessions from Guizhou, and seven accessions from Sichuan and Chongqing (Additional file 5: Table S5). To our knowledge, this is first comprehensive identification of a core germplasm based on a high-density SNP map at the scale of a large population, which is valuable for *P. massoniana* breeding practices.

**Table 3:** The allele proportion with sample size of masson pine

Sample size	Core SNP	Allele proportion(%)
3	49,393	52.44
4	58,568	62.18
6	68,768	73.01
8	74,939	79.56
10	78,766	83.62
20	82,003	87.06
40	85,455	90.72
61	85,844	91.14
81	87,448	92.84
122	89,761	95.29
163	91,333	96.96
183	92,030	97.70
204	94,194	100.00

### Population structure of the *P. massoniana* germplasm

**Fig. 2:**
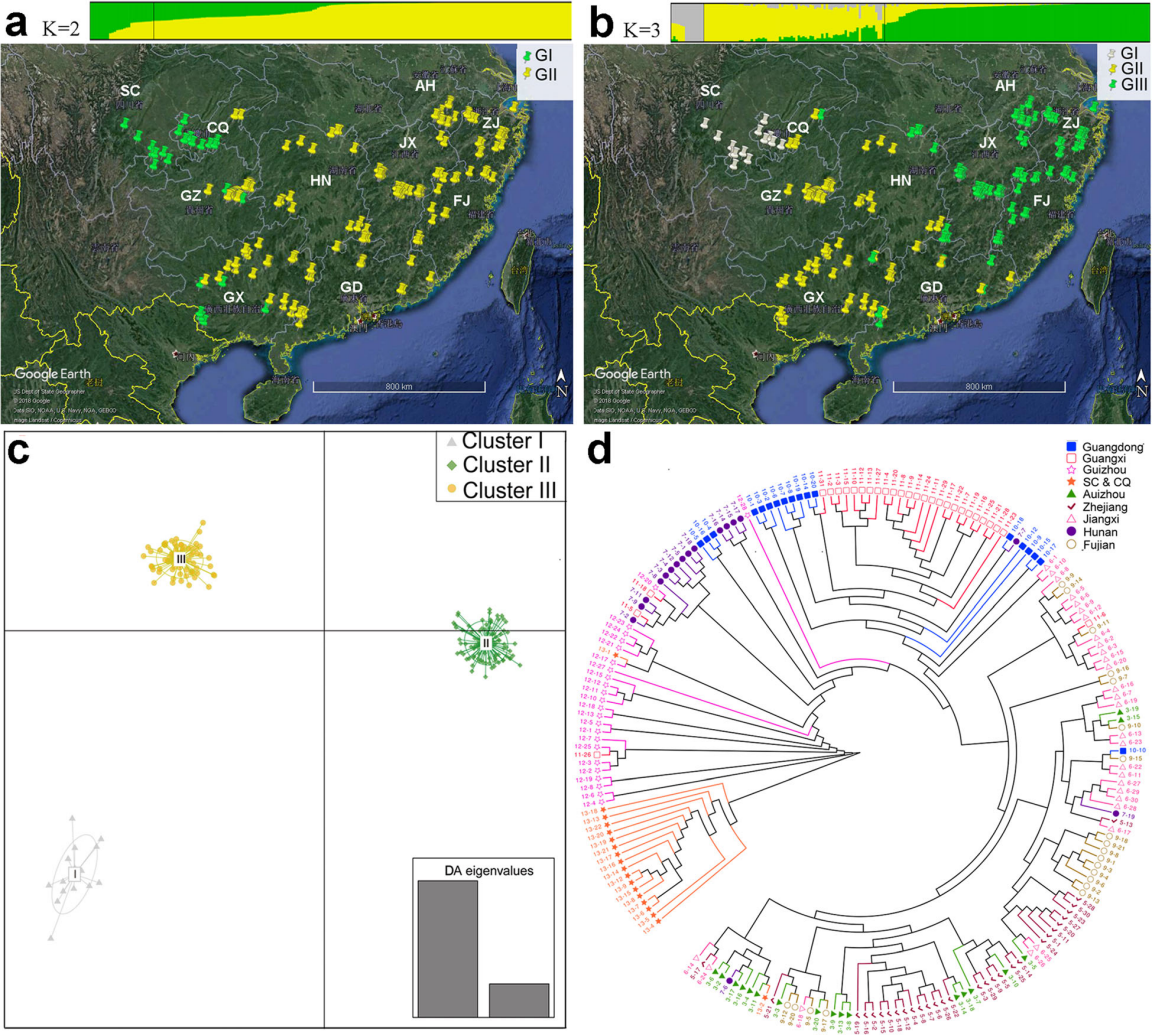
Characterization of the masson pine population. **a** and **b** Population structure and the corresponding groups of 204 masson pines. GI, Group I; GII, Group II; GIII, Group III. The image was created in Google Earth. **c** Two-dimensional discriminant analysis of principal component (DAPC) scatter plot. All genotypes were grouped in three clusters. Cluster I: Sichuan and Chongqing; Cluster II: Jiangxi, Fujian, Zhejiang, and Anhui; Cluster III: Guizhou, Guangxi, Guangdong, and Hunan. **d** Phylogenetic tree of the 204 masson pines based on the 94,194 SNPs. SC&CQ, Sichuan and Chongqing

To further understand the evolutionary history of masson pine, we used ADMIXTURE software [27] to estimate ancestry proportions for each accession. Genetic assignment analysis showed an optimal value of *K* = 2, which clearly separated the accessions of Chongqing and Sichuan from those of the other wild genotypes (Additional file 6: Fig. S1 and Additional file 7: Fig. S2A). The first group, which included mainly the clones from Chongqing and Sichuan provinces, had a high level of signals from the inter-population admixture (Fig. 2a). For *K = 3*, two new subpopulations from central southern China and southeastern China, arose from the accessions out of Chongqing and Sichuan (Fig. 2b). Notably, Group I, which included the clones from Chongqing and Sichuan provinces also showed high levels of admixture. Group II, which contained major clones from central southern China, including Guizhou, Guangxi, Guangdong, and Hunan provinces, showed an introgression signal from Group I. This was possibly contributed by natural hybridization occurring as a result of dispersion by animals or wind following separation. The clones from southeastern China, including Fujian, Jiangxi, Zhejiang, and Anhui provinces, were assigned to Group III. Interestingly, Group III kept their homogeneous genetic background, probably due to their geographical isolation, which blocks interspecific hybridization (Additional file 7: Fig. S2B). As expected, the population structure of masson pine genotypes is consistent with their geographical distributions.

Discriminant analysis of principal components (DAPC) revealed three genetic clusters driving the partitioning of diversity within our panel (Fig. 2c). Cluster I comprised only accessions from Sichuan and Chongqing provinces (94.4%) (Additional file 8: Table S6); Cluster II included mainly clones from Jiangxi (100%), Fujian (100%), Zhejiang (100%), and Anhui (100%) provinces, which contributed 88.3% to Cluster II; and Cluster III included major accessions from Guizhou (100%), Guangxi (96.6%), Guangdong (61.1%), and Hunan (88.2%) provinces. These three genetic clusters are geographically isolated. Cluster I consists of accessions living mainly in western China; the Cluster II accessions are distributed mainly in southeastern China; and Cluster III included accessions from central southern China. The results of the DAPC analysis were consistent with the population structure analysis using a value of *K =* 3.

We further estimated the genetic diversity of the different clusters. The genome-wide nucleotide diversity (π) of Cluster I (2.91 × 10^− 2^) was higher than those of Cluster II (2.77 × 10^− 2^) and Cluster III (2.83 × 10^− 2^) and exhibited the highest level of diversity. This result is also supported by the *H*_*e*_ value, which revealed the sequence diversity based on heterozygous sites (Table 4). The *Nei’s* genetic distance showed values ranging from 0.135 (Cluster II vs. Cluster III) to 0.303 (Cluster I vs. Cluster II), while the pairwise fixation index *F*_*st*_ ranged from 0.024 (Cluster II vs. Cluster III) to 0.110 (Cluster I vs. Cluster II), and the *Nei’s* and *F*_*st*_ genetic distances of Cluster I vs. Cluster II were higher than those of Cluster I vs. Cluster III (Additional file 9: Table S7). These observations suggest that masson pine of the Sichuang Basin has maintained a high genetic diversity and has a greater differentiation than that of southeastern China.

**Table 4:** Expected heterozygosity (*H*_*e*_) values of tree clusters inferred by DAPC

	*π*	*H*_*e*_
Cluster I	2.91 × 10^− 2^	0.3181
Cluster II	2.77 × 10^−2^	0.2928
Cluster III	2.83 × 10^−2^	0.2944

### Geographical origin and diffusion of the *P. massoniana* germplasm

**Fig. 3:**
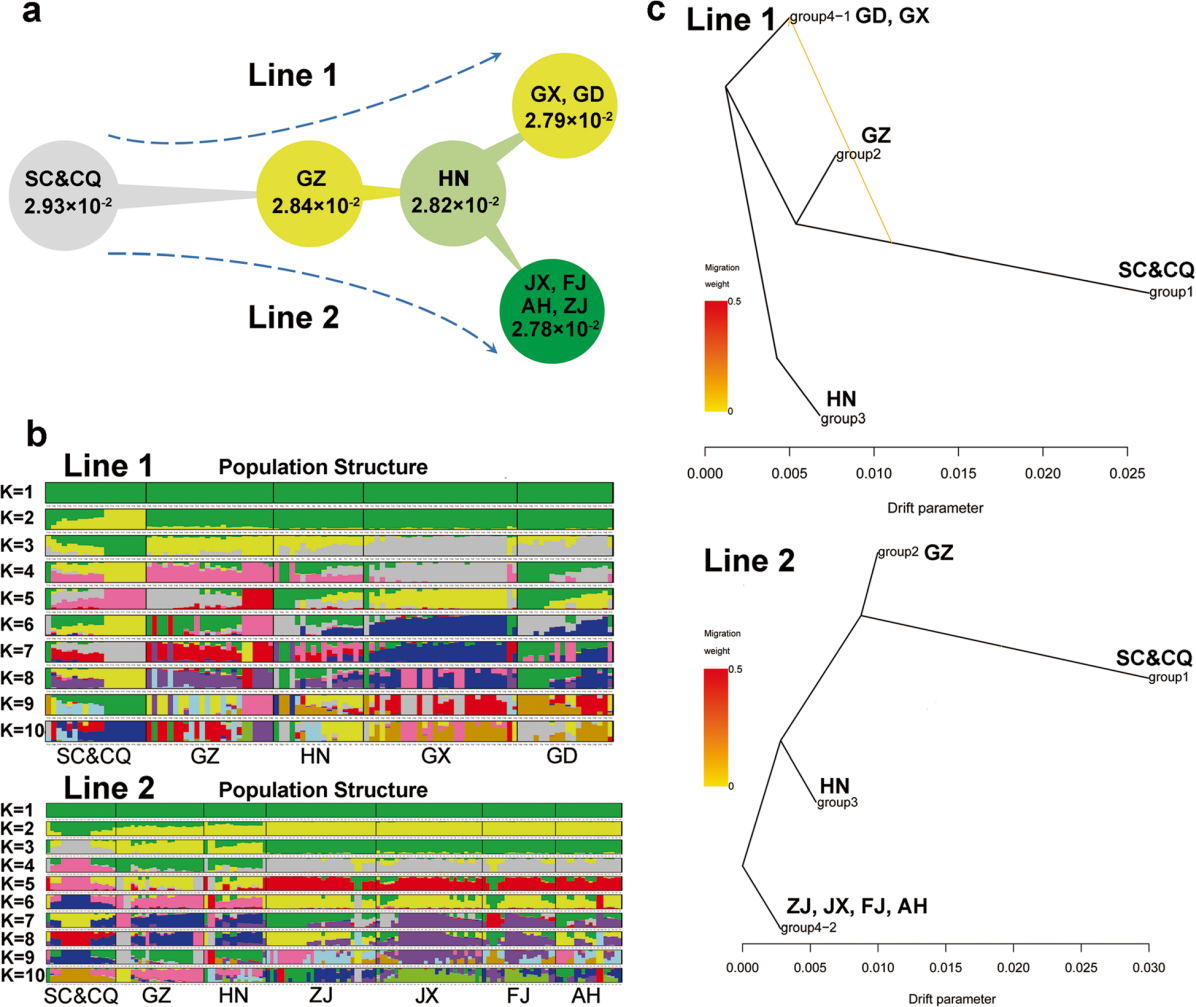
Spreading pathway of masson pine from the SC&CQ origin**. a** Orientation of two spreading lines in the masson pine evolution map. **b** Population structure of masson pines in the two spreading pathways. **c**Signals of introgression among different masson pine populations detected by the TreeMix program. SC&CQ, Sichuan and Chongqing; GZ, Guizhou; HN, Hunan; GX, Guangxi; GD,Guangdong; JX, Jiangxi; FJ, Fujian; AH, Anhui; ZJ, Zhejiang

To further elucidate the evolution map and spread pathway of masson pine, we examined the phylogeny of 204 masson pine genotypes by building a neighbor-joining phylogenetic tree (Fig. 2d). In addition, *P. taeda* was assigned as the outgroup of a maximum likelihood tree to identify the earliest diverging population, considered as the progenitor of the modern *P. massoniana* (Additional file 10: Fig. S3). The phylogenetic tree showed that the genotypes from the Sichuan Basin (Sichuang, Chongqing) were closest to those of *P. taeda* and were followed by other clades, suggesting that the Sichuan Basin is the geographic origin of masson pine. The Sichuan Basin was one of several glacial refuges for many species during the last Pleistocene glaciations [28, 29], which may have rescued the masson pine from an extinction event. The masson pine gradually migrated to the Guizhou Plateau after the end of the glacial epoch and gradually adapted to the plateau habitat. The genotypes of Hunan formed a subclade from a branch of the Guizhou clade and were followed by other masson pine genotypes of central southern China (Guangdong and Guangxi) and southeastern China (Jiangxi, Fujian, Zhejiang, and Anhui). It is notable that these genotypes clearly split into two subclades according to their geographical distributions. This observation allowed us to propose a hypothesis of two different orientation spreading lines in the masson pine evolution map (Fig. 3a). One migration line goes from Sichuan and Chongqing to Guizhou, then to Hunan, and then spreads into Guangdong and Guangxi. The other line goes from Sichuan and Chongqing to Guizhou, then to Hunan, and then spreads into Jiangxi, Fujian, Anhui, and Zhejiang. This hypothesis is strongly supported by the evidence for population structure (Fig. 3b). The population differentiation is significantly greater between Sichuang/Chongqing and Guizhou (*F*_*st*_ = 0.13) than between those of other populations, implying a strong variation in the genome for the new natural adaptation when first transferring from the basin to the plateau habitat. The nucleotide diversity is slightly higher for the progenitors of the Sichuang/Chongqing population (Fig. 3a). Signals of introgression between the populations for the two dissemination lines were detected by the TreeMix program. A hybridization signal from the Sichuan Basin population to the Guangdong/Guangxi population was detected (Fig. 3c).

### Associative transcriptomics and oleoresin yield

**Fig. 4:**
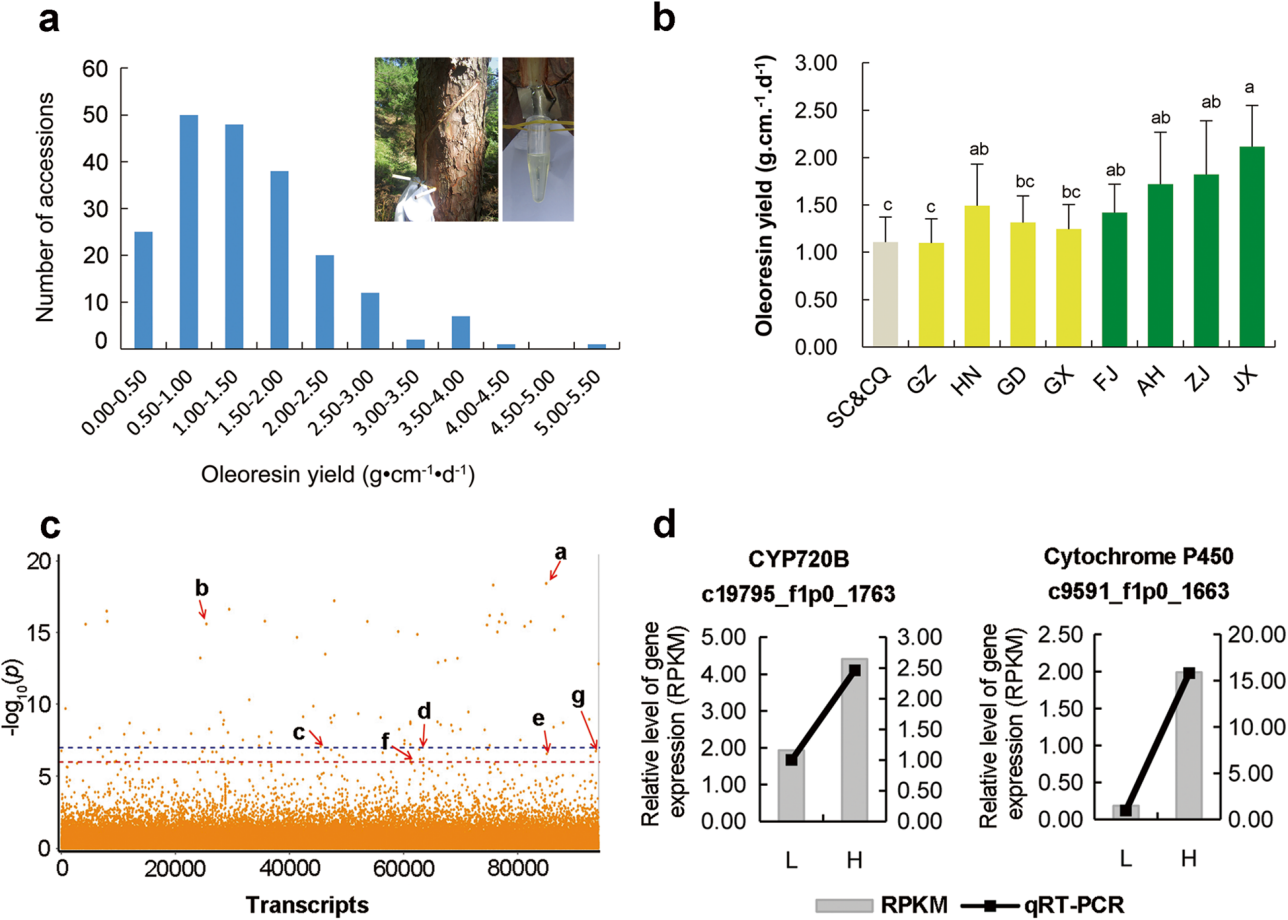
Discovery of the candidate genes associated with oleoresin yield by associative transcriptomics in masson pine. A, Distribution of oleoresin yield among 204 masson pines. B, Variation in oleoresin yield among nine populations. C, Manhattan plots resulting from the transcriptome-based association study data for oleoresin yield. a, Chitinase class I (c51955_f1p3_1546); b, Tubulin alpha chain (c20772_f1p4_1467); c, ABC transporter (c189021.graph_c0); d, AP2/ERF (c24091_f1p1_1286); e, AP2/ERF(c8825_f1p0_1733); f, CYP720B (c19795_f1p0_1763); g, Cytochrome P450 (c9591_f1p0_1663). D, Quantitative RT-PCR (qRT-PCR) validation of candidate genes associated with oleoresin yield

The oleoresin yield in the xylem of *P. massoniana* varied substantially in the 204 clones of masson pine, with levels varying from 0.00 to 6.07 g·cm^− 1^·d^− 1^ (Additional file 11: Table S8). The oleoresin yield distribution appeared to be positively skewed among the accessions (Fig. 4a). The yields of the accessions from the Sichuan Basin and Guizhou Province were significantly lower than that of the Hunan accessions (Fig. 4b). In the central southern China spreading path, the oleoresin yield reduced slightly when masson pine spread into Guangdong and Guangxi provinces, but was still higher than those of the accessions from the Sichuan Basin and Guizhou Province. In the southeastern China spreading path, the oleoresin yield significantly increased when masson pine spread into southeastern China, especially into Anhui, Zhejiang, and Jiangxi provinces.

Associative transcriptomics analysis identified 121 SNPs from 109 transcripts that were significantly associated with oleoresin yield at the *P* < 10^− 6^ significance level (Fig. 4c, Additional file 12: Fig. S4, and Additional file 13: Table S9). The most significant SNP (c51955_f1p3_1546, *R*^2^ = 0.51, *P* = 3.74E− 19) was localized in the transcript annotated as chitinase class I (Table 5). The mutated SNP occurred upstream of the coding region, but the expression of the transcript (c51955_f1p3_1546) was not significantly correlated with oleoresin yield.

**Table 5:** Candidate transcripts related to oleoresin yield in masson pine

Transcript ID	Pos	Pvalue	Marker R^2^	Allele	Gene annotation	Effect	Codon change
c51955_f1p3_1546	120	3.74E-19	0.51	C/G	Chitinase class I	Upstream	–
c20772_f1p4_1467	282	8.73E-16	0.29	G/A	Tubulin alpha chain	Nonsynonymous coding	cAc/cGc
c189021.graph_c0	317	8.79E-08	0.24	T/A	ABC transporter	Downstream	–
c24091_f1p1_1286	573	1.40E-07	0.19	A/G	AP2/ERF	Upstream	–
c8825_f1p0_1733	110	4.05E-07	0.21	T/C	AP2/ERF	Nonsynonymous coding	Tgc/Cgc
c19795_f1p0_1763	107	9.60E-07	0.19	T/C	CYP720B	Nonsynonymous coding	Ctc/Ttc
c9591_f1p0_1663	774	1.85E-07	0.20	C/T	Cytochrome P450	Synonymous coding	ctC/ctT

The family of CYP720B belonging to cytochrome P450 monooxygenases (P450), are an important class of enzymes involved in the biosynthesis of diterpene resin acids as the main content of oleoresin [30]. One CYP720B (c19795_f1p0_1763) and one cytochrome P450 (c9591_f1p0_1663) were found to be sequence-associated with the oleoresin yield (9.60E− 07, 1.85E− 07). The mutated SNP of CYP720B led to non-synonymous mutations, with the transition of codon CTC to TTC. The mutated SNP from cytochrome P450 (c9591_f1p0_1663) belongs to synonymous mutation. The expression of these two transcripts (c19795_f1p0_1763, c9591_f1p0_1663) was significantly correlated with oleoresin yield (*P* = 3.61E− 08, *P* = 2.13E− 08). The results of a quantitative real time polymerase chain reaction (qRT-PCR) using 10 high- and low-yielding oleoresin accessions showed higher expression levels for these two transcripts in high-yielding oleoresin masson pines (Fig. 4d).

The sequences of AP2 domain transcription factor and ABC transporter were associated with oleoresin yield in *P. taeda* [14]. In this study, two SNPs from AP2/ethylene-responsive transcription factors (ERFs) (c24091_f1p1_1286, c8825_f1p0_1733) and one SNP from ABC transporter (c189021.graph_c0) were also found to be significantly associated with oleoresin yield in masson pine. The SNP from AP2/ERF (c8825_f1p0_1733) resulted in non-synonymous coding and in the coding amino acid changing from cystine to arginine.

In addition, one SNP from the transcript of the tubulin alpha chain (c20772_f1p4_1467) was significantly associated with oleoresin yield in the sequence (*P* = 8.73E− 16) and expression level (*P* = 4.83E− 08) simultaneously. The SNP resulted in non-synonymous mutation, with the transition of codon CTC to TTC. However, the function of the tubulin alpha chain during the biosynthesis of oleoresin is unclear.

## Discussion

SNP markers have been used to evaluate diversity within many species, including *Populus trichocarpa* [31], *Vitis vinifera* [32], and *Ginkgo biloba* [33]. SNPs derived from transcriptome sequencing is a more efficient strategy for characterizing diversity in non-model or massive-genome species, since the sequences are detected on the coding regions rather than on the entire genome. In this study, 94,194 SNPs obtained by transcriptome sequencing were used to investigate the diversity of masson pine from ten provinces and municipalities in China. The *H*_*0*_ values (approximately 0.22) were slightly lower than the *H*_*e*_ values (0.30 across the populations), suggesting that frequent inbreeding events have occurred within the populations (Table 1). Either *H*_*e*_ or *H*_*0*_ can be used to assess genetic variation, but the *H*_*0*_ value is often influenced by the level of inbreeding within a population. Therefore, *H*_*e*_ is more often used in order to compare genetic diversity among different species, or of populations within the same species [3].

Masson pine has continuous native distributions in various regions of China. Hamrick et al. [34] found that the average expected heterozygosity within populations of tree species with widespread distributions was 0.228 using allozyme analyses. Huang and Zhang [35] reported that the *H*_*e*_ value of six natural populations of masson pine in Guizhou Province was 0.27, as determined by isozyme analysis. The genetic diversity of five populations of masson pine in Fujian Province was assessed, and the average value of *H*_*e*_ was found to be 0.22 [36]. In this study, the higher genetic variability detected might be attributed to the larger areas of the regions sampled, which covered almost the entire region that masson pine is native to. A similar outcome was observed for natural populations of Scots pine (*P. sylvestris*) [37, 38]. In addition, the differences in genetic diversity assessed in these studies could also be the result of different marker types, sampling locations, and sizes [39].

Both structure analysis and DAPC separated the Sichuan and Chongqing samples from the others (Fig. 2a, c). This differentiation was also in agreement with the values of *F*_*st*_ and *Nei’s* genetic distance (Additional file 9: Table S7), which revealed that the germplasm from Sichuan and Chongqing provinces had the highest values of *F*_*st*_ and *Nei’s* genetic distance, respectively, by DAPC. Although the structure analysis showed the minimum cross-validation error at *K* = 2, the cross-validation error at *K* = 3 was only slightly higher than that at *K* = 2. For *K* = 2, most of the germplasm from the other provinces not including Sichuan and Chongqing were grouped into one cluster (Fig. 2a). However, this cluster was divided into two groups for *K* = 3, which strongly corresponded with the clusters from DAPC, despite there being minor differences between the members of each cluster. The differentiation between Cluster II and Cluster III was relatively small, with *F*_*st*_ and *Nei’s* genetic distance values of 0.024 and 0.135, respectively, suggesting the masson pine germplasms from central southern and southeastern China are more closely related than that from southwestern China. In addition, compared to the other two clusters, Cluster I composed of Sichuan and Chongqing germplasm and located in southwestern China had the highest genetic diversity, with a *H*_*e*_ value of 0.318.

Climate is one of the main driving factors for adaptive evolution of forest trees [40, 41]. In the Northern Hemisphere, warm subtropical and temperate climates with rich gymnosperms in the Eocene turned into cold and strong seasonal climates from the Oligocene onwards over the Cenozoic in middle-latitude and high-latitude landmasses, especially in the Quaternary with large-scale ice cover and glaciations [42–44]. Many tree species became extinct as a result of the severe cold during this period. However, some tree species were better equipped to adapt to the sustained cooler conditions. In southern China, the complex topography helped numerous temperate forests survive the last glacial maximum in various “refugia” [45], such as *Ginkgo biloba*, *Metasequoia glyptostroboides*, *Glyptostrobus pensilis*, and *Liriodendron chinense*, all of which still survive in China. The Sichuan Basin, including the central and eastern parts of Sichuan Province and Chongqing Municipality, is surrounded by the Tibetan Plateau, the Dabashan Mountains, the Wushan Mountains, and the Yunnan–Guizhou Plateau (1000–3000 m above sea level). However, the elevation of the floor of the Sichuan Basin only ranges from 250 to 750 m. Although the glaciations also occurred in the Sichuan Basin during the Quaternary, the cooler climate did not lead to the extinction of plant and animal species, such as the surviving giant–panda (*Ailuropoda melanoleuca*). Therefore, the results suggest that the Sichuan Basin is one main refugium for many species [46]. These species were expanding to lower elevations during the glacial periods, and retreating to refugia at higher elevations during the interglacial stages [45, 47, 48].

To explore the evolutionary history of masson pine, we used loblolly pine (*P. taeda*) as a reference. Our study showed that the Sichuan Basin was the geographic origin of masson pine. This accords with previous results reported by Qin [46] from his study of the characteristics of both species’ needles. Structure analysis, however, showed two geographically distinct groups, and DAPC identified three clusters in this study, which suggest that the genes have been changing to adapt to the habitat.

Masson pine originally spread to Guizhou Province from the Sichuan Basin. Although neighboring Guizhou Province lies on the northern and western borders of Guangxi and Hunan provinces, respectively, masson pine only spread to Hunan from Guizhou subsequently. It might be that the Yunnan–Guizhou Plateau acted as the barrier hindering the spread of masson pine from Guizhou to Guangxi. Although Guangxi provenances were not highly distinguished from several other provenances using structure, DAPC, and cladogram analysis, the difference was significant between Guangxi and Guangdong provenances and the other provenances for growth traits. The spread of masson pine from the Guangxi and Guangdong provenances was faster, which is related to the thermal resources of those origins [7].

The breeding of masson pine can be accelerated by selecting those genes related to the target trait. *Np*ABC1 was reported to be the first transporter involved in the secretion of terpenoids in soybean [49]. In conifers, oleoresin is transported from living cells to resin ducts and flows from wounds when stems suffer abiotic stimuli [50]. Westbrook et al. [14] found that SNPs located in ABC transporters were associated with oleoresin yield, and inferred that ABC transporters participate in oleoresin transportation. In this study, the results of SNPs also indicated that ABC transporters were significantly associated with oleoresin yield, which suggests that ABC transporters may play an important role in regulating oleoresin yield by changing the sequences.

Chitinase plays a key role in modifying the structure of cell walls. Zhong et al. [51] found that the mutant gene of chitinase (*elp1*) would lead to lignin being ectopically deposited in the stems of *Arabidopsis* spp., and the walls of the lignified cells were not thickened. The function of chitinase might affect the transportation rate of oleoresin from living cells to resin ducts.

Most of the cytochrome P450s have been reported to be involved in the progress of secondary metabolism [52]. The CYP720B gene family of cytochrome P450s is specific for conifers, and can catalyze consecutive oxidation steps in the biosynthesis pathway of various diterpene resin acids as the main components of oleoresin [30]. We found that one SNP in CYP720B and one SNP in cytochrome P450 were significantly associated with oleoresin yield, and the SNP in CYP720B led to non-synonymous mutation. Therefore, the SNP in CYP720B was inferred to have an important influence on determining oleoresin yield by changing the sequence and expression level.

Ethylene can induce the biosynthesis and formation of traumatic resin ducts in many conifers [53]. AP2/ERF transcription factors are involved in the regulation of ethylene-responsive gene expression in the ethylene signaling pathway during abiotic stress. Over-expression of *OsEREBP1* belonging to the ERF family causes increased expression of genes related to lipid metabolism in rice [54]. In *P. taeda*, one SNP in the AP2 domain transcription factor was also associated with oleoresin yield [14], which was verified by our results for masson pine.

## Conclusions

It is important to understand the genetic architecture of masson pine in order to improve the oleoresin yield in the genetic breeding process. Although the genome of masson pine has not been sequenced, we obtained satisfactory results for genetic diversity, population structure, and trait–gene association based on 94,194 SNPs using the full-length transcriptome as a reference. Masson pine is clearly differentiated into two groups, and Sichuan and Chongqing provenance have been shown to be its geographical origin, from which masson pine diffuses outward along two distinct lines. Oleoresin yield exhibits two different trends along the two lines of diffusion and is associated with the genes of chitinase, CYP720B, cytochrome P450, ABC transporter, and AP2/ERF, some of which were also confirmed as being present in other conifers. The functions of these genes will be verified in future studies.

## Methods

### Sample collection

A clonal test of masson pine, located at Laoshan Forest Farm in the western part of Zhejiang Province, China (119°02′E, 29°33′N; altitude, 152 m above sea level), was used for this study. This trial included 400 clones (genotypes) obtained from 10 provinces and municipalities. In the 1980s, a national technical cooperation group for masson pine was established in China, and the scions for these clones were identified and provided by each provincial technical cooperation group authorized by the local government. Robust shoots as scions from wild trees were collected from the upper crowns of masson pine in April 1985. Subsequently, the scions were grafted onto two-year-old local seedlings of masson pine using the pith-cambium pairing grafting method and carried out by the Research Institute of Subtropical Forestry, Chinese Academy of Forestry, Hangzhou. In the following year, the clonal trail was established using these grafted seedlings to give a completely randomized design with 10 repetitions and 2.0 m × 3.0 m spacing between individual trees. Experimental research on these plants, including the collection of plant material, complied fully with institutional, national, and international guidelines. Field studies were conducted in accordance with local legislation. The authors complied with the Convention on International Trade in Endangered Species of Wild Fauna and Flora (CITES, also known as the Washington Convention, effective since 1975).

In this study, 204 healthy clones of masson pine from 10 provinces and municipalities of China were selected randomly (Additional file 14: Table S10). Before Chongqing became a Municipality in its own right in 1997, it was a city of Sichuan Province. When we collected scions from Sichuan and Chongqing, both situated in the Sichuan Basin, the analysis was carried out considering the germplasm from Chongqing and Sichuan as a single population.

Oleoresin yield was measured according to Liu’s method [55] between May and October, 2017 and 2018. The oleoresin yield of each tree was calculated as the yield of the individual tree per day per cm streak length in grams. Simultaneously, 5 mm of deep fresh secondary xylem tissues adjoining the cambium layer were harvested from the sample trees after removing the bark and phloem. These samples were placed in liquid nitrogen immediately in the field and then stored at − 80 °C for RNA extraction. These experiments were undertaken in the Research Institute of Subtropical Forestry, Chinese Academy of Forestry, Hangzhou.

### RNA extraction and PacBio-based sequencing

Total RNA from each sample was separately extracted and evaluated according to Liu’s method [56]. Briefly, total RNA from each sample was extracted using the Plant RNA kit RN38 EASYSpin plus (Aidlab Biotech, Beijing, China). The concentration and integrity of the total RNA was detected using an Ultraspec TM 2100 Pro UV/visible spectrophotometer and an Agilent 2100 Bioanalyzer. High-quality RNA samples were used to construct cDNA libraries. One microgram quantities of RNA from each sample were pooled together, and full-length cDNA was synthesized using the SMARTer™ PCR cDNA Synthesis Kit. The sizes of the full-length cDNAs were selected using BluePippin (SageScience, Beverly, MA, USA), and three libraries of differently sized cDNA (1–2 kb, 2–3 kb, and > 3 kb) were built. The size distribution of cDNA was then quantified using a Qubitfluorometer (Invitrogen, Thermo Fisher Scientific, Waltham, MA, USA), and the quality of the three libraries was assessed using the Agilent 2100 Bioanalyzer. Subsequently, SMRT sequencing was carried out using a Pacific Biosciences RS II (Menlo Park, CA, USA) platform at Biomarker Technologies Corporation, Beijing, China.

### Next-generation sequencing

The mRNA was obtained from high-quality total RNA for each sample using the magnetic beads enrichment procedure. Fragmentation buffer was used to fragment mRNA randomly. The first- and second-strand cDNA were synthesized. All of the cDNAs were purified using AMPure XP beads. After end repairing, adding A, and adaptor ligation, the fragment size of the purified cDNA was selected using the AMPure XP beads. The cDNA fragments were then enriched by PCR amplification, and the quality of the cDNA library for each sample was assessed using the Qubitfluorometer and the Agilent 2100 Bioanalyzer. Finally, the qualified cDNA library of each sample was paired-end sequenced on the Illumina HiSeq™ 2000 sequencing platform.

### Quality control of RNA-Seq data

Low-quality reads were filtered out based on the following four rules: (1) If one end of a pair-end read had > 5% “N” bases, then the pair-end read was removed; (2) For each pair-end read, if one of them had an average base quality less than 20, then they were both removed; (3) For each read, we trimmed its 3′ bases if their quality scores were less than 13. The trimming was stopped at the base with a quality score ≥ 13. Following trimming, if the number of remaining bases was less than 40, then the pair-end reads were removed; (4) Duplicates of pair-end reads were removed. Clean data were then used to call both SNPs and InDels.

### SNP and InDel calling

Filtered reads were then mapped to the reference sequences (full-length transcriptome) using the BWA-MEM algorithm of the Burrows Wheeler Aligner. SNPs were called using the Haplotype Caller in GATK across the 204 samples of masson pine. Finally, low-quality SNPs (QUAL < 30, MQ < 40.0, FS > 60.0, and QD < 2.0) were removed. InDels were called using the same pipeline as SNP calling. Raw InDels were filtered to reduce the false positives using GTAK variant filtration with the parameters: FS > 200, QD < 2, Read Pos Rank Sum < − 20.0.

### Genetic diversity analysis

The genetic parameters of observed heterozygosity (*H*_*0*_), expected heterozygosity (*H*_*e*_), minor allele frequency (MAF), and inbreeding coefficient (*F*) were estimated using PLINK software (version 1.9; http://zzz.bwh.harvard.edu/plink). Variation among populations, among clones within populations, and within clones was calculated via analysis of molecular variance (AMOVA) using Arlequin software (version 3.5.2; http://cmpg.unibe.ch/software/arlequin35).

### Phylogenetic analyses

For the phylogenetic tree, the genome of the loblolly pine (*P. taeda*) was first downloaded from the NCBI database (SRX4454630) and then aligned with the full-length transcriptome sequences. Subsequently, SNPs were called from the genome of the loblolly pine. Phylogenetic tree visualization and editing assignment were then performed using ITOL (http://itol.embl.de). The divergence time between masson pine and loblolly pine was obtained using the online Time Tree software (http://timetree.org). Finally, the divergence time for each germplasm was calculate and visualized using the MCMCtree program in the Phylogenetic Analysis by Maximum Likelihood (PML) package (http://abacus.gene.ucl.ac.uk/software/paml.html) [57].

### Population structure analyses

ADMIXTURE software (https://speciationgenomics.github.io/ADMIXTURE) [27] was used to visualize the genetic structure of the population. The preset ancestral population numbers ranged from *K* = 1 to *K* = 10. The most likely number of ancestral genetic groups was determined by the minimum *K* value on the cross-validation curve.

### Discriminant analysis of principal components (DAPC)

For DAPC, genetic data were first transformed into uncorrelated components using principal component analysis (PCA). The number of genetic clusters was then defined using k-means clustering, an algorithm that looks for the value of *K* that maximizes the variation between groups. The Bayesian information criterion (BIC) was calculated for *K* = 1–40, and the *K* value with the lowest BIC was selected as the optimal number of clusters. A discriminant analysis was then performed on the first 120 principal components using the function DAPC to efficiently describe the genetic clusters.

### Identification of genes associated with oleoresin yield

The association between SNPs and oleoresin yield was carried out using the mixed linear model (MLM) using Trait Analysis by aSSociation, Evolution and Linkage (TASSEL) (https://www.maizegenetics.net/tassel) [58]. The *P*-value corresponding to each association was calculated, and the association was significant when the *P*-value ≤1.06E− 5, which was estimated using 1/*n* named Bonferroni correction (*n* is the number of SNP markers).

### Quantitative qRT-PCR analysis

Ten high- and ten low-oleoresin-yielding RNA samples were used in qRT-PCR. The primer pairs (Additional file 15: Table S11) for seven genes of chitinase, tubulin alpha chain, AP2/ERF, ABC transporter, CYP720, the cytochrome P450 design, and the cDNA were amplified according to Liu’s method [56], and the expression levels of the genes were calculated using the 2^−ΔΔCt^ method [59]. Elongation factor 1-alpha (EF 1-alpha) was used to normalize the transcript profiles.

## Supplementary information

**Additional file 1 Table S1.** Statistical results following combination of full-length transcripts and unigenes of 204 accessions of masson pine

**Additional file 2 Table S2.** Numbers of reads mapped to reference sequences for 204 accessions of masson pine

**Additional file 3 Table S3.** SNPs and InDels obtained using transcriptomes of 204 accessions

**Additional file 4 Table S4.** Effect of SNP mutation for each unigene

**Additional file 5 Table S5.** Core accessions with 90.7 and 95% allele proportions

**Additional file 6 Figure S1.** Cross-validation error rate for each K value

**Additional file 7 Figure S2.** Population structure and the corresponding groups of 204 masson pines when *K* = 2 and *K* = 3.

**Additional file 8 Table S6.** Three genetic clusters identified by DAPC based on SNP markers

**Additional file 9 Table S7.** *Nei’s* and *F*_*st*_ genetic distance calculated for three clusters inferred by DAPC

**Additional file 10 Figure S3.** Phylogenetic relationships among a masson pine population and *P. taeda* as the outgroup

**Additional file 11 Table S8.** Oleoresin yield in the xylem of 204 clones of masson pine

**Additional file 12 Figure S4.** Quantile-quantile plots resulting from the transcriptome-based association study data for oleoresin yield

**Additional file 13 Table S9.** SNP markers significantly associated with variation in oleoresin yield in masson pine

**Additional file 14 Table S10.** Origin of the 204 clones of masson pine

**Additional file 15 Table S11.** Primers designed from the sequences of the transcriptome library in masson pine using Primer Premier 3.0

## Data Availability

All data generated or analyzed during this study are included in this published article (and its additional information files). All sequencing data generated in this study are available from the SRA Archive (https://www.ncbi.nlm.nih.gov/sra) under BioProject ID: PRJNA636925. The raw sequences of one full-length transcriptome on PacBio SMRT Sequencing platform were deposited in the NCBI GeneBank under SRA run accessions SRR11912706. The RNA-seq raw sequences of 204 samples on the Illumina HiSeqTM 2000 sequencing platform were deposited in the NCBI GeneBank under SRA run accessions SRR11912579- SRR11912705 and SRR11912707- SRR11912783.

## Abbreviations

AMOVA: Analysis of molecular variance
AP2: APETALA2 domain transcription factor
DAPC: Discriminant analysis of principal components
ERFs: Ethylene-responsive transcription factors
NGS: Next-generation sequencing
qRT-PCR: Quantitative reverse transcription-polymerase chain reaction
SNP: Single-nucleotide polymorphisms
