# Supplementary figures and images for: A transcriptomic variation map provides insights into the genetic basis of Pinus massoniana Lamb. evolution and the association with oleoresin yield
Source: BMC Plant Biol. 2020 Aug 13;20:375. doi: 10.1186/s12870-020-02577-z (PMC7427074; doi:10.1186/s12870-020-02577-z)

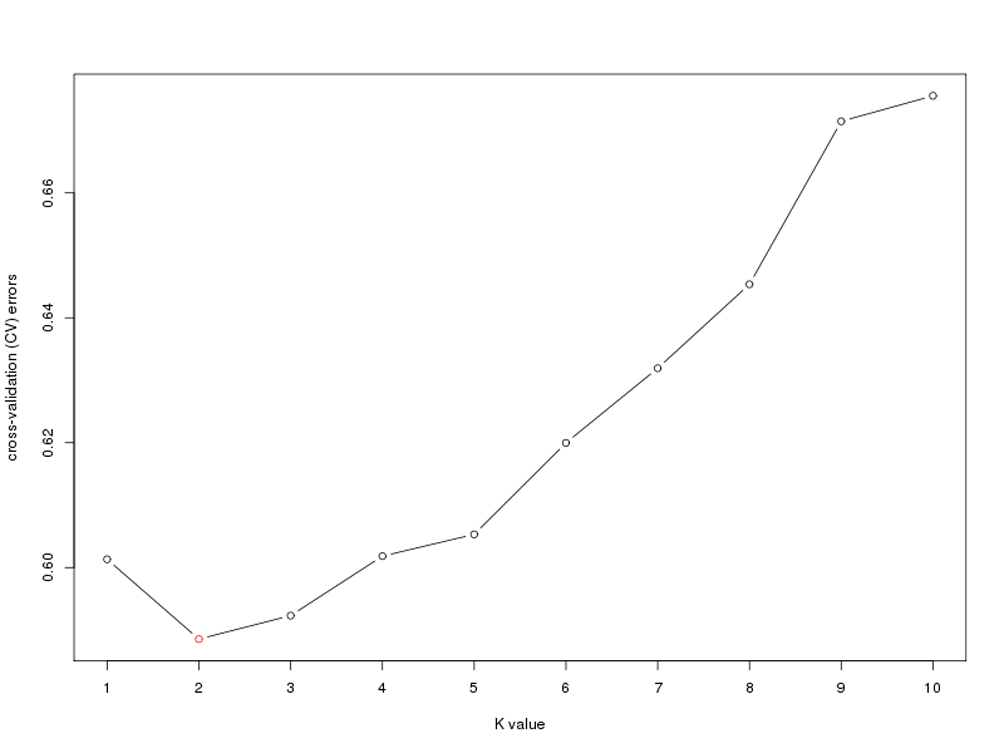

Supplement: Supplementary file 6 — Additional file 6 Figure S1. Cross-validation error rate for each K value [file 12870_2020_2577_MOESM6_ESM.tif]

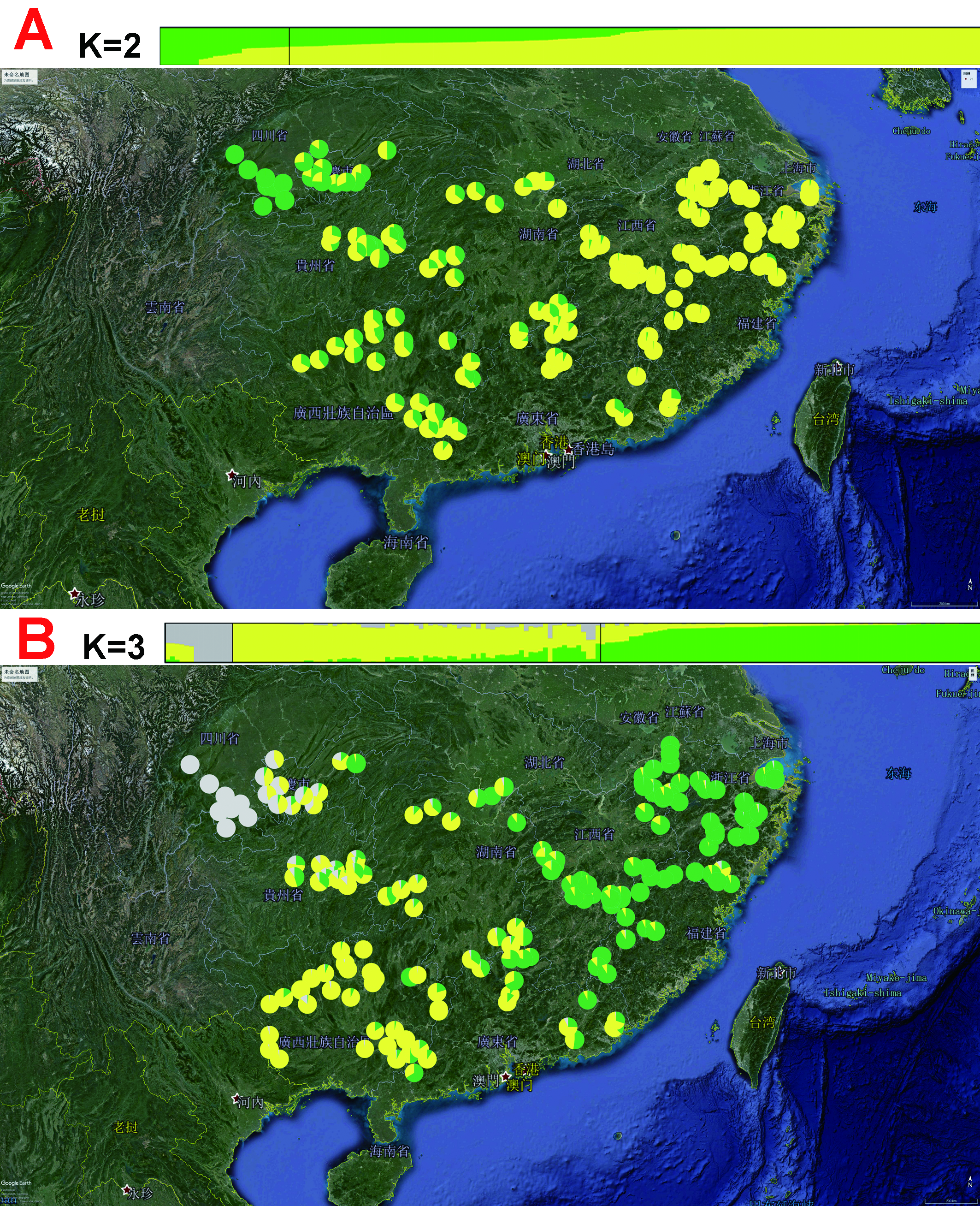

Supplement: Supplementary file 7 — Additional file 7 Figure S2. Population structure and the corresponding groups of 204 masson pines when K = 2 and K = 3. [file 12870_2020_2577_MOESM7_ESM.tif]

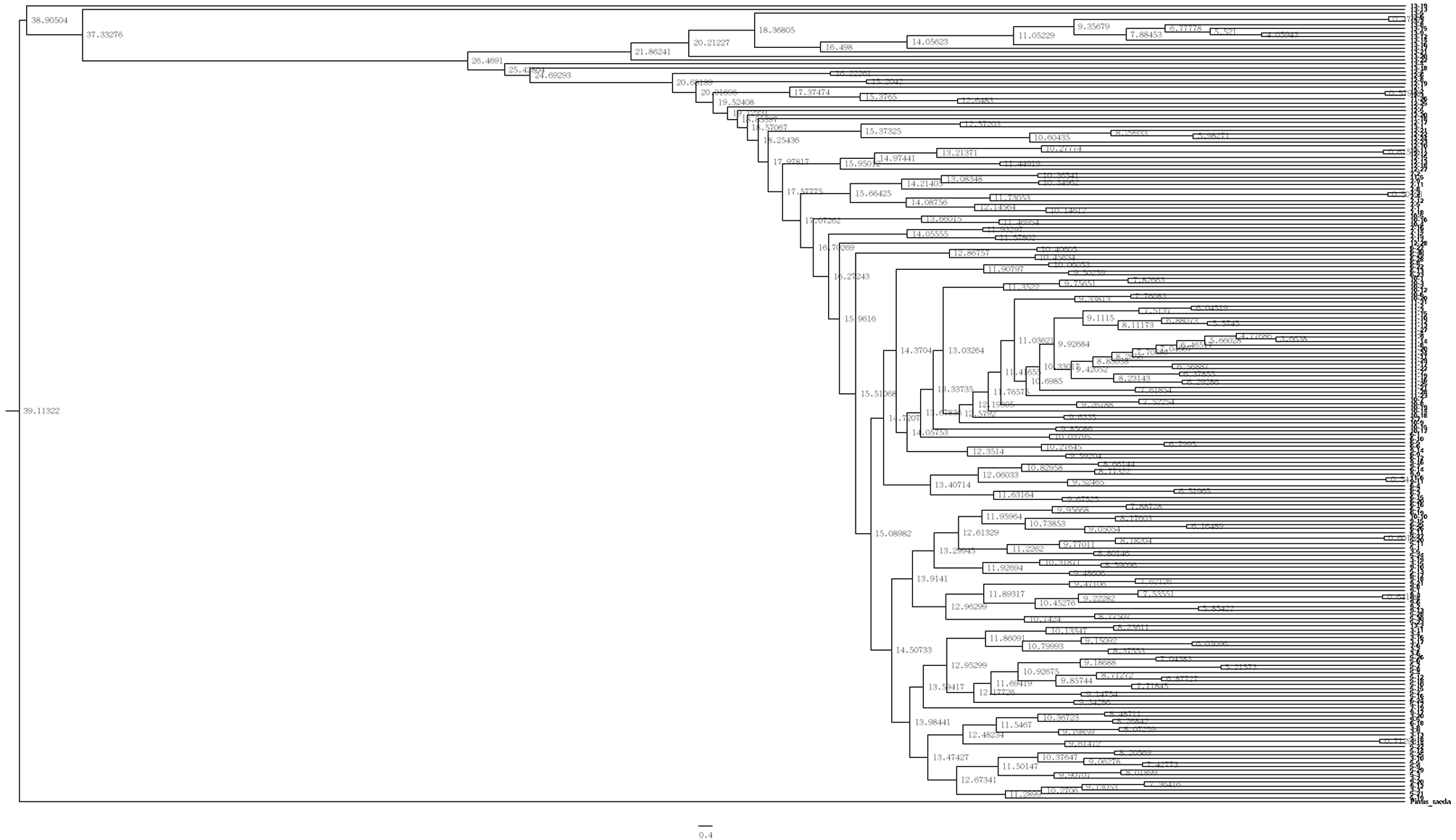

Supplement: Supplementary file 10 — Additional file 10 Figure S3. Phylogenetic relationships among a masson pine population and P. taeda as the outgroup [file 12870_2020_2577_MOESM10_ESM.tif]

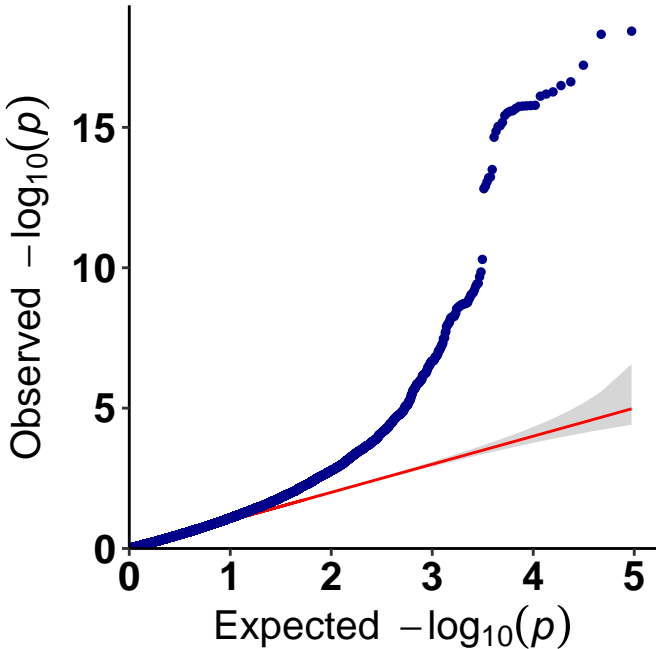

Supplement: Supplementary file 12 — Additional file 12 Figure S4. Quantile-quantile plots resulting from the transcriptome-based association study data for oleoresin yield [file 12870_2020_2577_MOESM12_ESM.pdf]
