# Supplementary material for: A transcriptomic variation map provides insights into the genetic basis of Pinus massoniana Lamb. evolution and the association with oleoresin yield
Source: BMC Plant Biol. 2020 Aug 13;20:375. doi: 10.1186/s12870-020-02577-z (PMC7427074; doi:10.1186/s12870-020-02577-z)
Supplement: Supplementary file 9 — Additional file 9 Table S7. Nei’s and Fst genetic distance calculated for three clusters inferred by DAPC [file 12870_2020_2577_MOESM9_ESM.docx]

**Table S7** *Nei’s* and *F_st_* genetic distance calculated for three clusters inferred by DAPC.

|  | Cluster I | Cluster II | Cluster III |
| --- | --- | --- | --- |
| Cluster I |  | 0.110 | 0.074 |
| Cluster II | 0.303 |  | 0.024 |
| Cluster III | 0.268 | 0.135 |  |
