# Supplementary material for: A transcriptomic variation map provides insights into the genetic basis of Pinus massoniana Lamb. evolution and the association with oleoresin yield
Source: BMC Plant Biol. 2020 Aug 13;20:375. doi: 10.1186/s12870-020-02577-z (PMC7427074; doi:10.1186/s12870-020-02577-z)
Supplement: Supplementary file 15 — Additional file 15 Table S11. Primers designed from the sequences of the transcriptome library in masson pine using Primer Premier 3.0 [file 12870_2020_2577_MOESM15_ESM.docx]

**Table S11.** Primers designed from the sequences of the transcriptome library in masson pine using Primer Premier 3.0

| Genes | Forward primer | Reverse primer |
| --- | --- | --- |
| Chitinase (c51955_f1p3_1546) | 5'-GCGGTGGGTTTCTGGGATTA-3' | 5'-TGTAGCACAAACCCCAAGCA-3' |
| ABC transporter (c189021.graph_c0) | 5'-CCTTGAAACGAGTGTGCACC-3' | 5'-TCTTCACCCCACAGCAACAA-3' |
| CYP720B (c19795_f1p0_1763) | 5'-TCCATTTCTCAACCACGCCA-3' | 5'-CGTTCCGTGGAGCTTCTTCT-3' |
| Cytochrome P450 ( c9591_f1p0_1663) | 5'-AGAGACAACTGCGATGGACA-3' | 5'-GATCACCGTGTCCATCTCCT-3' |
| AP2 / ERF (c24091_f1p1_1286) | 5'-CCAAGGGTTTCGAGCTTCCT-3' | 5'-TACCAAGCCAAACCCTAGCG-3' |
| AP2/ERF ( c8825_f1p0_1733) | 5'-AGGGATGCAAGCAGAAGAGT-3' | 5'-ATCTCTAATCTCCGCAGCCC-3' |
| Tubulin alpha chain ( c20772_f1p4_1467) | 5'-ACTTGGTCCCGTATCCTCGA-3' | 5'-CAGCGGCATTGACATCCTTG-3' |
| EF 1- alpha | 5'-CTGCGATGTCCCTCATGTTA-3' | 5'- AACAAGGTCTTTCCCCTCGT-3' |
